# Acute Myocardial Infarction: Molecular Pathogenesis, Diagnosis, and Clinical Management

**DOI:** 10.1002/mco2.70418

**Published:** 2025-10-07

**Authors:** Mengmeng Zhu, Yiwen Li, Qian Xu, Wenting Wang, Yanfei Liu, Yue Liu

**Affiliations:** ^1^ National Clinical Research Center for Cardiovascular Diseases of Traditional Chinese Medicine Xiyuan Hospital of China Academy of Chinese Medical Sciences Beijing China; ^2^ Beijing Key Laboratory of Traditional Chinese Medicine Basic Research on Prevention and Treatment for Major Diseases Experimental Research Center China Academy of Chinese Medical Sciences Beijing China; ^3^ Department of Traditional Chinese Medicine Peking Union Medical College Hospital Chinese Academy of Medical Sciences and Peking Union Medical College Beijing China; ^4^ The Second Department of Geriatrics Xiyuan Hospital China Academy of Chinese Medical Sciences Beijing China; ^5^ Key Laboratory of Disease and Syndrome Integration Prevention and Treatment of Vascular Aging Xiyuan Hospital of China Academy of Chinese Medical Sciences Beijing China

**Keywords:** acute myocardial infarction, inter‐organ crosstalk, myocardial ischemia/reperfusion infarction, pathogenesis, ventricular remodeling

## Abstract

Acute myocardial infarction (AMI) is a cardiovascular disease characterized by myocardial necrosis resulting from acute coronary artery occlusion. Although standardized diagnostic and therapeutic protocols have markedly reduced its mortality, AMI remains a leading cause of death and disability worldwide. Contemporary AMI research has evolved from an initial focus on local myocardial injury to a broader perspective encompassing the entire disease course, including tissue damage, remodeling, and multi‐organ interactions. This review systematically delineates the key molecular mechanisms underlying AMI and subsequent ventricular remodeling, while also exploring the complex interplay between the heart and other organs such as the gut, brain, kidney, and liver. From a clinical standpoint, we summarize the historical evolution of AMI diagnostic criteria and management strategies, highlighting current classification systems, novel diagnostic technologies, and the integration of artificial intelligence tools. Furthermore, we present recent evidence‐based advances in established therapeutic approaches, along with emerging strategies ranging from cellular to genetic interventions. Future directions aim to integrate mechanistic insights with interdisciplinary clinical strategies to establish a systematic and precision‐based framework for AMI prevention and management.

## Graphical abstract

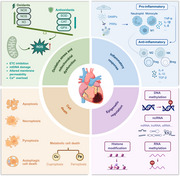

## 1 Introduction

**FIGURE 1:**
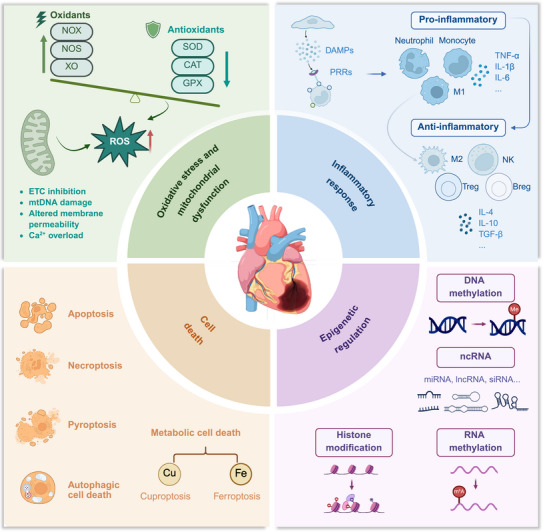
Key pathogenic mechanisms in AMI. Oxidative stress and mitochondrial dysfunction induce ROS accumulation, while DAMPs‐PRRs interaction triggers inflammatory cascades. Programmed cell death pathways and epigenetic regulation collectively constitute the core components of AMI pathogenesis. AMI, acute myocardial infarction; Breg, regulatory B cells; CAT, catalase; DAMPs, damage‐associated molecular patterns; ETC, electron transport chain; GPX, glutathione peroxidase; lncRNAs, long non‐coding RNAs; miRNAs, microRNAs; mtDNA, mitochondrial DNA; ncRNA, non‐coding RNAs; NK, natural killer; NOX, NADPH oxidase; NOS, nitric oxide synthase; PRRs, pattern recognition receptors; ROS, reactive oxygen species; SOD, superoxide dismutase; Treg, regulatory T cells; XO, Xanthine oxidase.

Acute myocardial infarction (AMI) is a major cardiovascular disease that seriously endangers human health. It is characterized by the sudden occlusion of coronary arteries caused due to the rupture or erosion of atherosclerotic plaques, which leads to severe myocardial ischemia and hypoxia and eventually results in irreversible death of myocardial cells [1]. Over the past two decades, the age‐adjusted mortality rate (AAMR) of AMI has declined at an average annual rate of 4.6%, a trend likely attributable to advances in early diagnostic technologies, improvements in reperfusion therapies, and a substantial increase in the use of guideline‐recommended medications [2]. Nevertheless, post‐AMI complications, particularly adverse ventricular remodeling and heart failure following ischemia/reperfusion (I/R) injury, continue to be major contributors to long‐term mortality and disability in patients [3, 4]. Therefore, a deeper understanding of the molecular basis of AMI pathogenesis is crucial for identifying potential therapeutic targets. In recent years, research on the mechanisms of AMI has gradually expanded from the simple ischemic stage to the entire process including myocardial ischemia/reperfusion injury (MIRI), myocardial repair, and ventricular remodeling, involving complex and interrelated multiple mechanisms such as oxidative stress and mitochondrial dysfunction [5], inflammatory responses [6], cell death [7], and epigenetic regulation [8] (Figure 1). Furthermore, recent studies have also discovered complex interactions between AMI and other organs, leading to the occurrence of systemic complications and worsening the prognosis of patients.

The understanding of AMI diagnosis and treatment has undergone lengthy development. In terms of diagnosis, it has evolved from solely relying on symptom assessment to integrating electrocardiograms (ECGs) and myocardial enzyme tests along with coronary intervention for visualized diagnostics. The current research direction is focused on refining the diagnostic subtypes [9] and is attempting to incorporate more sensitive biomarkers, advanced imaging techniques [10], and artificial intelligence (AI) technologies [11] to enhance the precision and non‐invasiveness of diagnosis. There has also been a significant shift in the focus of treatment in clinical management strategies. The era of simple symptomatic treatment and standardized drug therapy has been replaced by an era dominated by coronary revascularization and is further breaking through into emerging precision medicine fields such as myocardial cell regeneration [12] and gene intervention [13].

This article reviews the latest research progress in the fields of molecular mechanisms, diagnostic methods and clinical management of AMI, aiming to comprehensively expound the mechanism and current status of diagnosis and treatment of AMI, with the expectation of optimizing clinical decision‐making for AMI and providing reference basis for future research directions.

## 2 Molecular Pathogenesis

The molecular mechanisms of AMI involve oxidative stress, mitochondrial dysfunction, inflammatory cascade reactions, and the coordinated activation of multiple programmed cell death pathways, all of which are modulated by epigenetic regulatory mechanisms. These processes collectively drive myocardial injury and constitute the core pathological components of AMI pathogenesis. On this basis, cell death induced by AMI serves as the initiating factor of ventricular remodeling, which in turn triggers sustained inflammation, fibrosis, angiogenesis, and neuroendocrine activation, collectively driving the progression of ventricular remodeling after AMI.

### 2.1 Oxidative Stress and Mitochondrial Dysfunction

Under physiological conditions, reactive oxygen species (ROS) acts as a subcellular signaling molecule to regulate processes such as myocardial cell proliferation, migration, and apoptosis. Oxidative systems including NADPH oxidase (NOX) and nitric oxide synthase (NOS) maintain a dynamic balance with antioxidant systems such as superoxide dismutase (SOD), catalase (CAT), and glutathione peroxidase (GPX) [14]. During AMI, this balance is disrupted, leading to excessive ROS accumulation that triggers oxidative stress responses. Acting in concert with mitochondrial dysfunction, these events drive the progression of cardiomyocyte and myocardial tissue injury.

In the acute ischemic phase, interruption of oxygen supply to the myocardium results in impaired mitochondrial electron transport chain (ETC) function, leading to the overproduction of superoxide anions. Moreover, cardiomyocytes in different myocardial regions display distinct metabolic profiles: the infarct core and border zone undergo a metabolic shift from predominant fatty acid oxidation (FAO) to compensatory glycolysis, whereas the remote myocardium remains primarily FAO‐dependent [15, 16]. This ischemia‐induced metabolic reprogramming enhances glycolytic flux, leading to lactate accumulation and intracellular acidosis. Although acidosis transiently inhibits the opening of the mitochondrial permeability transition pores (mPTPs), it simultaneously activates the Na^+^/H^+^ exchanger (NHE) and Na^+^/Ca^2+^ exchanger (NCX), thereby increasing intracellular Na⁺ and Ca^2^⁺ concentrations [17, 18, 19]. The abnormal expression of multiple regulatory factors further exacerbates oxidative stress. For example, tripartite motif‐containing protein 21 (TRIM21) increases ROS production by activating the nuclear factor kappa B (NF‐κB)/NOX2 pathway [20], while neuraminidase (NEU1) aggravates mitochondrial energy metabolic collapse by suppressing the sirtuin‐1 (SIRT1)/peroxisome proliferator‐activated receptor γ coactivator α (PGC‐1α) axis and reducing mitochondrial DNA (mtDNA) stability [21]. Additionally, as AMI progresses, the significant downregulation of key antioxidative stress factors also impairs the intrinsic antioxidant capacity. For instance, decreased expression of mitochondrial histidine triad nucleotide‐binding protein 2 (HINT2) compromises its ability to maintain nicotinamide adenine dinucleotide (NAD) homeostasis and to enhance the antioxidant functions of SOD and glutathione (GSH) [22]. Similarly, reduced fibroblast growth factor 7 (FGF7) expression inhibits the phosphatidylinositol‐3‐kinase α (PI3Kα)/AKT pathway, preventing nuclear translocation of nuclear factor erythroid 2‐related factor 2 (Nrf2) and disrupting the mitochondrial localization of the glycolytic rate‐limiting enzyme hexokinase II (HXK2). These effects collectively reduce the expression of antioxidant proteins such as CAT and SOD‐2, as well as the disruption of mitochondrial homeostasis, resulting in the generation of a large amount of ROS and exacerbating oxidative stress damage [23].

Reperfusion therapy aims to restore myocardial blood flow; however, the abrupt transition from ischemia to reperfusion can paradoxically trigger or exacerbate myocardial injury, a phenomenon known as MIRI. During this process, the restoration of blood flow is accompanied by a surge of oxygen molecules into the previously ischemic tissue, and the sudden reintroduction of oxygen is a critical pathological driver of sharply elevated oxidative stress. The influx of oxygen interacts with ischemia‐accumulated succinate during reoxygenation, generating a burst of ROS via reverse electron transport (RET) through mitochondrial complex I. Excessive ROS then induce mitochondrial dysfunction through lipid peroxidation [24, 25]. Concurrently, rapid shifts in intra‐ and extracellular pH further aggravate Ca^2^⁺ overload, while ROS act synergistically with Ca^2^⁺ to promote sustained opening of the mPTP. This process exacerbates mitochondrial impairment and establishes a vicious cycle of ROS overproduction [26, 27]. I/R also induces upregulation of S100A8/A9, which inhibits the PGC‐1α/ nuclear respiratory factor 1 (NRF1) signaling pathway through activation of the toll‐like receptor 4 (TLR4)/Erk pathway, thereby downregulating NDUF gene expression and consequently reducing the activity of mitochondrial complex I in the ETC, which further amplifies mitochondrial dysfunction [28]. The ROS burst directly activates NF‐κB signaling, with its subunit p65 recruiting histone deacetylase 3 (HDAC3) to form a complex with the antioxidant transcription factor Nrf2, antagonizing the Nrf2‐ antioxidant responsive element (ARE) antioxidant pathway and further weakening the antioxidant system [29]. Additionally, reperfusion enhances Ca^2+^ influx mediated by the mechanically sensitive channel Piezo1, activating Calpain, further increasing Drp1 phosphorylation and its translocation to the mitochondria, leading to excessive mitochondrial fission and dysfunction [30]. Elevated intracellular Ca^2+^ also induces the upregulation of the calcium‐binding protein S100A16, which inhibits the calcium/calmodulin‐dependent protein kinase 2 (CAMKK2)/AMP‐activated protein kinase (AMPK) pathway by binding to calmodulin (CaM), resulting in decreased antioxidant capacity, ROS accumulation, and exacerbated inflammatory responses, thus aggravating MIRI [31].

### 2.2 Inflammatory Response

The inflammatory response in AMI exhibits a dynamic, multi‐stage evolution, involving the complex regulation of both innate and adaptive immune cells. The pathological process begins with the activation of an acute innate immune response triggered by plaque rupture or erosion. Necrotic myocardial cells release damage‐associated molecular patterns (DAMPs), such as high mobility group box 1 (HMGB1), heat shock proteins (HSP), and S100A8/A9, which interact with pattern recognition receptors (PRRs) like toll‐like receptors (TLRs), nod‐like receptor pyrin domain 3 (NLRP3) inflammasome, and receptor for advanced glycation end‐products (RAGE). This interaction triggers the release of pro‐inflammatory cytokines and chemokines, including IL‐1β, IL‐6, and tumor necrosis factor (TNF)‐α, thereby initiating an inflammatory cascade and recruiting monocytes, macrophages, and neutrophils to the infarct area [32].

In the early stages of infarction, pro‐inflammatory Ly6C^hi^ monocyte subsets dominate, later differentiating into macrophages. B lymphocytes further promote the recruitment of Ly6C^hi^ monocytes to the infarct zone by secreting chemokines such as CCL7 [33]. This pro‐inflammatory microenvironment drives macrophages toward classical M1 polarization. M1 macrophages, while clearing apoptotic cells and necrotic tissue debris, also release pro‐inflammatory cytokines (e.g., IL‐1β, TNF‐α), chemokines, and matrix metalloproteinases (MMPs), thereby expanding the extent of ischemic injury [34, 35]. Recent studies have identified a critical role for caspase‐recruitment domain‐containing domain family member 9 (CARD9) in this process. CARD9 expression in macrophages is markedly upregulated 1 day after MI and peaks at Day 3. Through activation of the NF‐κB signaling pathway, CARD9 induces the expression of the lipid‐binding protein lipocalin‐2 (LCN2). LCN2, upon binding to its receptor SLC22A17, significantly enhances the secretion of MMP9 by macrophages, exacerbating post‐MI cardiac dysfunction [36]. Meanwhile, complement activation products C3a/C5a and ELR⁺ CXC chemokines work in concert to drive the recruitment of neutrophils to the infarct area. After reaching a peak at 24 h, the infiltrating neutrophils exacerbate tissue damage by forming neutrophil extracellular traps (NETs) [37]. The traditional view posits that the elevation of chemokines stimulates the release of neutrophils from the bone marrow, serving as the primary mechanism underlying the early surge of neutrophils following AMI. However, recent studies have revealed that endothelial cells release endothelial cell‐derived extracellular vesicles (EC‐EVs) carrying vascular cell adhesion molecule‐1 (VCAM‐1) and miRNA‐126 during the early ischemic phase. These EC‐EVs can rapidly mobilize neutrophils from the spleen to the peripheral blood, thus representing an additional critical pathway contributing to the early increase in neutrophils [38]. In addition, neutrophils regulate their intravascular retention via a circadian “aging” program. The core clock gene Bmal1 drives CXCL2 expression, which induces neutrophil aging through the CXCL2/CXCR2 signaling axis. This leads to the daytime accumulation of neutrophils in blood vessels and triggers thromboinflammatory responses that are independent of NETs, thereby exacerbating I/R injury. These findings provide novel mechanistic insights into the circadian susceptibility of AMI and suggest that tailoring treatment regimens according to the circadian time window may represent a promising strategy to reduce I/R injury after AMI [39].

As the disease progresses to the late stage of AMI, the infarcted area gradually enters the repair phase. Activated regulatory T cells (Tregs) play a crucial role in suppressing excessive inflammation and promoting macrophage polarization toward the anti‐inflammatory M2 phenotype, resulting in the production of anti‐inflammatory cytokines such as IL‐4, IL‐10, and TGF‐β [40]. Exogenous administration of Tregs can markedly increase the expression of cardiac repair–associated factors such as nidogen‐1 and IL‐10, while reducing CD8^+^ T‐cell infiltration and the presence of pro‐inflammatory Ly6C^hi^ CCR2^+^ monocytes/macrophages. This intervention promotes the polarization of macrophages toward reparative subtypes, ultimately enhancing cardiac repair. In addition, neutrophils have also been found to participate in the late stage of AMI by inducing macrophage polarization toward an M2‐like phenotype [41]. Regulatory B cells (Bregs) contribute to improved ventricular remodeling by attenuating C–C motif chemokine receptor 2 (CCR2)‐mediated mobilization of monocytes from the bone marrow and their recruitment into the infarcted myocardium, thereby limiting the influx of inflammatory cells [42]. Meanwhile, natural killer (NK) cells promote cardiac repair by releasing IL‐10 and other mediators that suppress local inflammation, acting synergistically with other immune regulatory pathways [43]. It is noteworthy that even after the acute inflammatory response has subsided, residual inflammatory risk (RIR), which is a persistent low‐grade chronic inflammatory state, remains closely associated with an increased risk of subsequent cardiovascular events. Investigating the potential regulatory mechanisms underlying RIR is therefore an important direction for future research [44].

### 2.3 Cell Death

**FIGURE 2:**
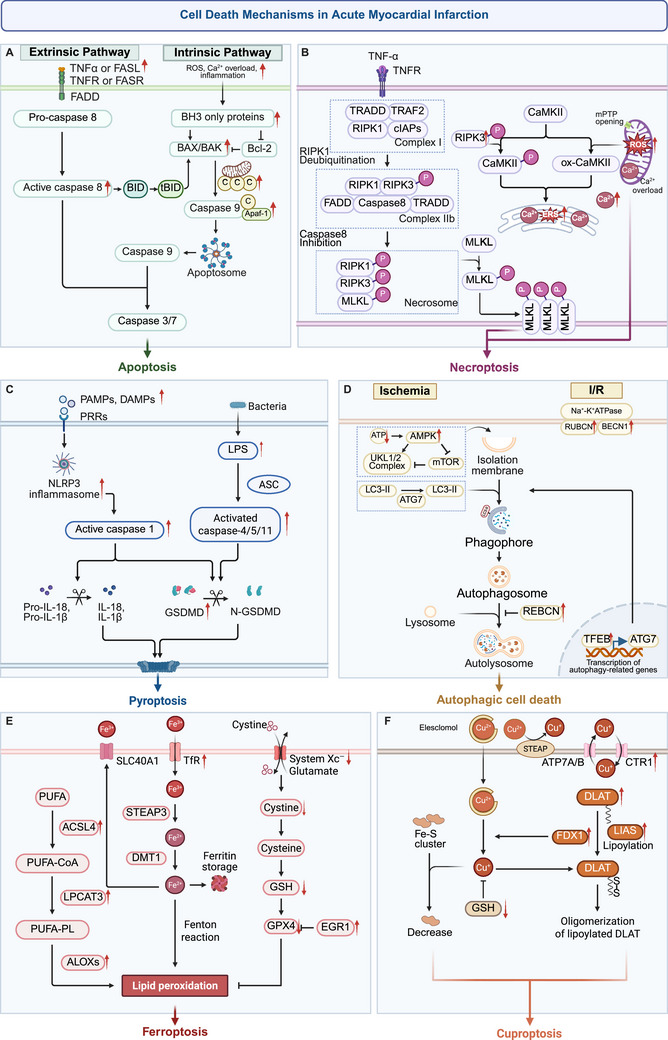
Cell death mechanisms in AMI. (A) Apoptosis is primarily activated by endogenous factors and can also be initiated through the binding of death ligands to their receptors. (B) Necroptosis is induced by TNF signaling upon caspase‐8 inhibition, leading to MLKL‐mediated pore formation via the RIPK1‐RIPK3 phosphorylation cascade, culminating in osmotic lysis. (C) Pyroptosis proceeds through both classical and non‐classical pathways, causing GSDMD cleavage and the release of mature IL‐18 and IL‐1β. (D) Autophagic cell death is induced under ischemic conditions via AMPK‐mediated inhibition of mTOR, promoting protective autophagy; during I/R injury, it is driven by increased expression of BECN1 and RUBCN proteins, leading to excessive autophagy. (E) Ferroptosis is marked by lipid peroxidation and dysfunction of System Xc‐, (F) whereas cuproptosis involves enhanced intracellular copper influx, resulting in the aggregation of toxic proteins. ACSL4, acyl‐CoA synthetase long‐chain family member 4; AMI, acute myocardial infarction; Apaf‐1, apoptotic protease‐activating factor 1; BECN1, beclin 1; CaMKII, calcium/calmodulin‐dependent protein kinase II; cIAP1/2, cellular inhibitor of apoptosis protein 1/2; DLAT, dihydrolipoamide S‐acetyltransferase; FDX1, ferredoxin 1; GPX, glutathione peroxidase; GSDM, gasdermin; LPCAT3, lysophosphatidylcholine acyltransferase 3; LPS, lipopolysaccharide; MLKL, mixed lineage kinase domain‐like protein; mPTP, mitochondrial permeability transition pore; PAMPs, pathogen‐associated molecular patterns; PL‐PUFA, polyunsaturated‐fatty‐acid‐containing phospholipid; PUFA, polyunsaturated fatty acids; PUFA‐CoA, PUFA‐acylated AA; RIPK1, receptor‐interacting serine/threonine‐protein kinase 1; RIPK3, receptor‐interacting serine/threonine‐protein kinase 3; ROS, reactive oxygen species; RUBCN, rubicon; TFEB, transcription factor EB; TfR1, transferrin receptor 1; TRAF, TNFR‐associated factor; TRADD, TNFR1‐associated death domain.

Cell death can be categorized into accidental cell death (ACD) and regulated cell death (RCD). ACD is a passive process triggered by external factors such as physical or chemical injury, whereas RCD is a highly controlled and orderly process governed by a series of intracellular signaling pathways and molecular mechanisms. Traditional RCD includes apoptosis, pyroptosis, and autophagic cell death, while metabolic cell death is a new form of RCD, including ferroptosis and cuproptosis. Different types of RCD display distinct morphological, biochemical, and molecular characteristics (Table 1). In the context of AMI, multiple RCD pathways are activated in a coordinated manner, collectively driving myocardial injury (Figure 2).

**TABLE 1:** Comparison of features and regulatory mechanisms in different forms of regulated cell death.

Features	Apoptosis [50, 51, 56, 57, 58, 59]	Necroptosis [62, 63, 64]	Pyroptosis [70, 74, 75, 76, 77]	Autophagic cell death [79, 80, 81]	Ferroptosis [85, 86]	Cuproptosis [97]
Morphological features	Plasma membrane blebbing, cell shrinkage, nuclear fragmentation, apoptotic body formation	Cell swelling, plasma membrane rupture, contents release	Cell swelling, cell membrane rupture, formation of membrane pores	Autolysosome formation, cell vacuolization	Mitochondrial shrinkage, increased mitochondrial membrane density, mitochondrial cristae disruption	Mitochondrial shrinkage, cell membrane rupture, ER stress, chromatin damage
Biochemical features	Caspase activation, DNA fragmentation	Activation of RIPK1, RIPK3, MLKL	GSDMD‐NT release, IL‐1β/IL‐18 secretion	Increased lysosomal activity	Iron accumulation, lipid peroxidation, GSH depletion, GPX4 reduction	Copper accumulation, lipoylated proteins aggregation
Triggering mechanisms	Intracellular signals (intrinsic pathway)/death receptor (extrinsic pathway) activation	Death receptor activation	Inflammasome activation (classical)/LPS (non‐classical)	Excessive autophagy induction	System Xc⁻ inhibition or TfR1 activation (exogenous)/antioxidant enzyme inhibition (endogenous)	Copper ion accumulation and increased lipoylation of TCA cycle enzymes
Key regulatory molecules	Bcl‐2 family (anti‐apoptotic: e.g., Bcl‐2, Bcl‐xL; pro‐apoptotic effectors: e.g., BAX, BAK); caspase‐3/7/8/9	RIPK1, RIPK3, MLKL	Caspase1/4/5/11, GSDMD, NLRP3, IL‐1, IL‐1β	Beclin1, LC3, Rubicon, TFEB	GPX4, ACSL4, SLC7A11	FDX1, DLAT, CTR1, lipoylated proteins
Inhibitors	Z‐VAD‐FMK and so forth	Nec‐1, NSA, and so forth	VX‐765, Disulfiram, and so forth	3‐MA, CQ, and so forth	Liproxstatin‐1, ferrostatin‐1, and so forth	TTM, and so forth

Abbreviations: 3‐MA, 3‐methyladenine; CQ, chloroquine; GSDMD, gasdermin D; MLKL, mixed lineage kinase domain‐like protein; Nec‐1, necrostatin‐1; NSA, necrosulfonamide; RIPK1, receptor‐interacting serine/threonine‐protein kinase 1; RIPK3, receptor‐interacting serine/threonine‐protein kinase 3; TTM, tetrathiomolybdate.

#### 2.3.1 Apoptosis

Apoptosis is an important form of cell death in AMI and is coordinated by the intrinsic pathway (mitochondrial pathway) and the extrinsic pathway (death receptor pathway) [45]. In cases of myocardial ischemia or I/R, increased mitochondrial ROS, Ca^2+^ overload, acidosis, and inflammatory response activation collectively trigger the intrinsic apoptotic pathway [46, 47, 48, 49]. In this pathway, Bcl‐2 family proteins play a central regulatory role. Pro‐apoptotic BH3‐only proteins (such as PUMA, BID, and BAD) neutralize anti‐apoptotic proteins (such as Bcl‐2 and Bcl‐xL) or directly activate pro‐apoptotic effector proteins (such as BAX and BAK). This leads to the oligomerization of BAX/BAK and their integration into the outer mitochondrial membrane, forming mitochondrial outer membrane permeabilization (MOMP) pores, which in turn facilitates the release of cytochrome C into the cytoplasm [50]. Cytochrome C binds to apoptotic protease‐activating factor 1 (Apaf‐1) to form apoptosomes that activate caspase‐9 and subsequently to activate cascade effector caspases‐3/7, ultimately inducing apoptosis [51]. In addition, myocardial ischemia or I/R can also activate the transcription factor p53, upregulating the expression of pro‐apoptotic genes like BAX and PUMA to accelerate the apoptotic process [52, 53]. Myocardial hypoxia, through upregulating large tumor suppressor kinase 2 (Lats2) levels, induces excessive mitochondrial fission, resulting in abnormal opening of the mPTP and mitochondrial membrane rupture. Consequently, mtDNA leaks into the cytoplasm, activating the cGAS/STING/p65 signaling pathway and ultimately inducing cardiomyocyte apoptosis [54]. At the same time, hypoxia also promotes the upregulation of tripartite motif‐containing 55 (Trim55) expression, further accelerating cardiomyocyte apoptosis by inhibiting the Nrf2/HO‐1 pathway [55]. Recent research has shown that nuclear factor erythroid 2‐related factor 3 (Nfr3) is highly expressed in the infarct border zone of AMI, exacerbating excessive accumulation of mitochondrial ROS and amplifying apoptotic signals by inhibiting paired‐like homeodomain transcription factor 2 (Pitx2) expression [46]. The extrinsic pathway is triggered by death ligands such as Fas ligand (FASL) and TNF binding to cell surface death receptors Fas receptor (FASR) and TNF receptor (TNFR). During AMI, the levels of death ligands such as FASL and TNF‐α are significantly elevated, forming death‐inducing signaling complexes (DISCs) by binding to Fas receptors or TNFR1, recruiting adaptor protein FADD, and activating caspase‐8 [56, 57, 58, 59]. Upon activation, caspase‐8 not only directly activates downstream effector caspases‐3/7 to drive the extrinsic apoptotic pathway but also cleaves BID into tBid, thereby triggering the intrinsic pathway. This dual function facilitates cross‐talk and synergistic amplification between extrinsic and intrinsic apoptotic signaling [60, 61].

#### 2.3.2 Necroptosis

The core molecular mechanism of necroptosis relies on the receptor‐interacting serine/threonine‐protein kinase1 (RIPK1)/receptor‐interacting serine/threonine‐protein kinase 3 (RIPK3)/mixed lineage kinase domain‐like protein (MLKL) signaling axis, with its activation being regulated by the TNFR1 signaling complex [62, 63]. Upon binding of TNF‐α and other death receptor ligands to TNFR1, RIPK1 forms complex I with TNFR1‐associated death domain (TRADD), TNFR‐associated factor (TRAF), and cellular inhibitor of apoptosis protein 1/2 (cIAP1/2). Deubiquitination of RIPK1 facilitates the dissociation of TRADD and RIPK1 from complex I, enabling RIPK1 to interact with RIPK3 via the RIP homotypic interaction motif (RHIM) domain to assemble the necrosome. This process triggers MLKL phosphorylation and oligomerization, ultimately leading to disruption of cell membrane integrity [64]. However, during the myocardial ischemia stage, research has found that RIPK3 directly triggers necroptosis of cardiomyocytes through a RIPK1‐independent pathway [65]. Studies on I/R have also shown that RIPK3 not only directly phosphorylates the Thr287 site of calcium/calmodulin‐dependent protein kinase II (CaMKII) but also induces oxidative modification of Met281/282 via activation of NOX. These dual actions synergistically promote the opening of the mPTP and calcium overload, thereby triggering MLKL‐independent necroptosis in cardiomyocytes [66]. Furthermore, RIPK3 has been identified as a DAMP that is released extracellularly. Upon binding to the RAGE, it activates CaMKII signaling, thereby amplifying necroptosis and exacerbating inflammation, which in turn intensifies I/R injury. These findings highlight the multifaceted roles of RIPK3 both within and outside the cell [67]. Recent studies have also discovered that I/R can upregulate TRPC6, mediating calcium influx and CaMKII phosphorylation, forming a pro‐necroptotic Ca^2^⁺/CaMKII signaling cascade, exacerbating oxidative stress and inducing necroptosis [68]. In addition, necroptosis‐associated molecules in non‐myocardial cells can exhibit different regulatory mechanisms. Specifically, MLKL in macrophages mediates inflammatory adhesion via integrin αvβ1, thereby contributing to segmental myocardial necrosis. Notably, this process can occur independently of RIPK3, offering a novel cross‐cellular perspective on the necroptosis mechanism in AMI [69].

#### 2.3.3 Pyroptosis

Pyroptosis is a form of inflammatory cell death marked by plasma membrane pore formation mediated by the gasdermin (GSDM) family of proteins upon inflammasome activation. This process can be categorized into the classical and non‐classical pathways [70, 71]. The classical pathway is mediated by inflammasomes. Danger signals such as pathogen‐associated molecular patterns (PAMPs) and DAMPs activate PRRs such as NLRP1, NLRP3, and NLRC4, initiating the assembly of the inflammasome with adaptor protein ASC and procaspase‐1 [72, 73]. Activated caspase‐1 not only cleaves GSDMD to release its N‐terminal domain (GSDMD‐NT), which mediates plasma membrane pore formation but also processes pro‐IL‐1β and pro‐IL‐18 into mature IL‐1β and IL‐18, respectively. These mature cytokines are subsequently released via the GSDMD‐induced pores. The non‐classical pyroptosis pathway is independent of PRR and is directly activated by lipopolysaccharide (LPS) via caspase‐4/5/11, which subsequently cleaves GSDMD to induce plasma membrane pore formation. In AMI, the synergistic activation of both classical and non‐classical pathways further exacerbates myocardial injury. During MI, the transcription factor interferon regulatory factor2 (IRF2) is upregulated, directly binding to the GSDMD promoter to drive its transcription and promote the expression of pyroptosis‐related proteins such as GSDMD and c‐caspase1 [74]. Further studies have demonstrated that in hypoxia/reoxygenation and high‐glucose conditions, cardiomyocytes activate ROS‐dependent NLRP3 inflammasomes via LPS signaling. This activation significantly elevates the levels of IL‐1β and IL‐18, thereby exacerbating pyroptosis and aggravating myocardial injury [75]. In addition to direct activation of NLRP3, ubiquitination modifications also play an important role in the regulation of pyroptosis during I/R. During I/R, the expression of E3 ubiquitin ligase membrane‐associated RING finger protein 2 (MARCH2) is upregulated. MARCH2 catalyzes the K48‐linked ubiquitination of phosphoglycerate mutase 5 (PGAM5), promoting its proteasomal degradation. This disrupts the liquid–liquid phase separation (LLPS)‐mediated condensate formation between PGAM5 and mitochondrial antiviral signaling protein (MAVS), thereby inhibiting MAVS‐dependent recruitment and activation of the NLRP3 inflammasome [76]. Another E3 ubiquitin ligase, tripartite motif containing 16 (TRIM16), directly ubiquitinates NLRP3 to promote its degradation, thereby alleviating I/R injury [77]. The non‐classical pyroptosis pathway also plays a critical role in myocardial injury. Under hypoxia/reoxygenation conditions, cardiomyocytes experience oxidative stress, which activates caspase‐11. Caspase‐11 directly cleaves GSDMD to produce GSDMD‐NT, leading to the formation of membrane pores, cell membrane rupture, and the specific release of IL‐18. Notably, this pathway does not depend on the maturation of IL‐1β [78].

#### 2.3.4 Autophagic Cell Death

Autophagic cell death is a form of programmed cell death triggered by the excessive activation of autophagy [79]. Notably, autosis represents a distinct subtype of autophagic cell death that is regulated by Na^+^‐K ^+^‐ATPase and characterized by unique morphological and biochemical features [80]. Autophagic cell death presents a dual mechanism in the development of AMI [81]. During myocardial ischemia, energy metabolism disorders activate AMPK, inhibiting the downstream mTOR signaling pathway to induce protective autophagy, maintaining myocardial cell energy homeostasis for survival. Inhibiting autophagic activity during ischemia with 3‐MA or AMPK inhibitors can significantly exacerbate myocardial cell death, indicating the core protective role of AMPK‐dependent autophagy during myocardial ischemia. In contrast, during I/R, AMPK is no longer activated but rather stimulates excessive autophagy by increasing Beclin 1 (BECN1) expression, resulting in cardiomyocyte death. MI Further research has shown that I/R‐induced oxidative stress can upregulate BECN1 expression and overactivate autophagy by activating the p53/myogenin axis, while the cardiac autophagy inhibitor factor (CAIF) can directly bind to the p53 protein to inhibit cardiac autophagy and alleviate MI [82]. Meanwhile, the Rubicon (RUBCN) protein was also found to be significantly upregulated in the late stage of I/R, blocking the fusion of autophagosomes and lysosomes, leading to the accumulation of autophagosomes [83]. Furthermore, studies have demonstrated that during the late phase of I/R, the transcription factor EB (TFEB) is persistently activated and translocates to the nucleus. Through transcriptional regulation, it upregulates the expression of autophagy‐ and lysosome‐related functional genes such as BECN1, ATG7, and LAMP1. This synergizes with RUBCN‐mediated autophagic flux blockage, leading to excessive accumulation of autophagosomes and ultimately resulting in autosis. Targeted knockdown of RUBCN or TFEB can significantly reduce the accumulation of autophagosomes and mitigate cardiomyocyte autosis, thereby significantly reducing I/R injury [80, 84]. However, long‐term inhibition of TFEB may worsen infarct size by interfering with the early protective autophagy, suggesting that interventions targeting TFEB should be time‐specific. Therefore, further research is necessary to explore the role of autophagy at different stages of MI.

#### 2.3.5 Metabolic Cell Death

Metabolic cell death is triggered by imbalances in the metabolism of certain nutrients or metals. In recent years, ferroptosis and cuproptosis have been the most extensively studied forms of metabolic cell death in the context of AMI. Ferroptosis is a type of iron‐dependent cell death caused by the abnormal accumulation of lipid peroxidation products at the cell membrane, which can be initiated through both exogenous (transporter‐dependent) and endogenous (enzyme‐regulated) pathways [85, 86]. The exogenous pathway primarily initiates through the suppression of membrane transport proteins such as the cystine/glutamate antiporter (System Xc^−^) or activation of iron transport proteins. During reperfusion, downregulation of the SLC7A11 subunit in the antioxidative system of System Xc^−^ suppresses its function, reducing cysteine uptake and GSH synthesis, while high expression of p53 further inhibits SLC7A11 [87]. GSH is continuously released to the extracellular space through multidrug resistance protein 1 (MRP1), leading to GSH depletion within cell [88]. Meanwhile, 4‐hydroxy‐2‐nonenal (4‐HNE) accumulates during I/R, directly binding to the cysteine residues of GPX4 and OTU5, promoting GPX4 ubiquitination and degradation [89]. Additionally, during I/R, ubiquitin‐specific protease 7 (USP7) stabilizes and activates p53 through deubiquitination, further upregulating the expression of transferrin receptor 1 (TfR1), enhancing iron uptake, and inducing iron overload [90]; DNA methyltransferase 1 (DNMT‐1) promotes the expression of nuclear receptor coactivator 4 (NCOA4) through epigenetic regulation, facilitating ferritinophagy [91, 92]. Ultimately, it intensifies lipid peroxidation and inhibits the activity of GPX4, exacerbating myocardial injury. The endogenous pathway primarily characterized by the inhibition of the key antioxidant enzyme GPX4 and the increased synthesis of polyunsaturated fatty acid phospholipids (PUFA‐PLs) mediated by acyl‐CoA synthetase long‐chain family member 4 (ACSL4) and lysophosphatidylcholine acyltransferase 3 (LPCAT3), both of which synergistically contribute to the accumulation of lipid peroxides. In the early stages of myocardial ischemia, early growth response‐1 (EGR‐1) is significantly upregulated, inhibiting the antioxidant function of GPX4. Ischemic signals simultaneously induce the upregulation of ALOX15 expression, specifically catalyzing the peroxidation of PUFA‐PL, promoting lipid peroxidation and damaging cell membrane function, thereby driving ferroptosis [93, 94, 95]. In addition, during reperfusion, the activity of ALOX15 continuously increases and generates the key metabolite 15‐HpETE. 15‐HpETE degrades Pgc1α through ubiquitination, leading to mitochondrial lipid peroxidation, dysfunction and morphological abnormalities, further promoting ferroptosis of cardiomyocytes and exacerbating MIRI [96].

Copper ion homeostasis is essential for maintaining normal cellular functions. Cuproptosis, a novel form of cell death dependent on mitochondrial respiration, primarily involves the uptake and accumulation of copper ions via specific transporters (e.g., CTR1/SLC31A1) and their subsequent interaction with key molecules involved in mitochondrial metabolism [97]. During AMI, overexpression of CTR1 significantly enhances the influx of Cu^+^ into cardiomyocytes. The core regulatory protein ferredoxin1 (FDX1) in cuproptosis promotes lipoic acid synthase (LIAS)‐dependent protein lipoylation, driving direct binding of Cu^2^⁺ to the dihydrolipoamide S‐acetyltransferase (DLAT) subunit of the pyruvate dehydrogenase complex and inducing its abnormal oligomerization. This aggregation disrupts mitochondrial iron–sulfur (Fe‐S) cluster protein function and impairs the tricarboxylic acid (TCA) cycle, ultimately triggering cell death [98, 99, 100]. Moreover, bioinformatics analysis further reveals the role of cuproptosis‐related genes (CRGs) in AMI. GLS was identified as a biomarker of high diagnostic value, showing an association with immune and hypoxic pathways. DLST, GZMA, GIMAP5/6/7, and TRAF3IP are significantly related to both immune cell infiltration and cuproptosis [101, 102, 103]. However, most current findings are based on bioinformatics predictions. Future studies should focus on further experimental validation to elucidate the underlying mechanisms and causal relationships of CRGs in AMI.

### 2.4 Epigenetic Regulation

Epigenetic mechanisms, including DNA methylation, RNA methylation, histone modifications, and the regulation by non‐coding RNAs (ncRNAs; such as microRNAs [miRNAs] and long ncRNAs [lncRNAs]), play critical roles in the pathophysiology of AMI.

#### 2.4.1 DNA Methylation

DNA methylation is primarily regulated by the DNMT family and typically occurs at cytosine‐phosphate‐guanine (CpG) dinucleotides. This modification can directly interfere with the binding of specific transcription factors or indirectly recruit methyl‐CpG‐binding proteins (MeCPs), which act as transcriptional repressors by altering chromatin structure and silencing gene expression [104, 105]. In the context of AMI, DNA methylation exhibits distinct temporal dynamics and functional relevance. Genome‐wide changes in methylation profiles occur as early as 6 h after AMI onset, coinciding with large‐scale alterations in gene expression, suggesting that methylation serves as a key regulatory mechanism during the early phase of AMI [106]. During the I/R phase, the expression and activity of DNMT1 are significantly upregulated, leading to enhanced global methylation. This suppresses genes involved in mitochondrial function and exacerbates myocardial ischemic injury [107]. Studies on blood samples from AMI patients have identified significant changes in methylation levels at multiple CpG sites. For example, hypermethylation of the ABCA1 promoter impairs reverse cholesterol transport, accelerating atherosclerosis progression [108]. Hypermethylation of the Treg‐specific demethylated region (TSDR) in the forkhead Box P3 (FOXP3) gene has been identified as an independent predictor of poor prognosis in acute coronary syndrome (ACS), potentially due to reduced anti‐inflammatory function of Tregs, thereby promoting atherosclerotic progression [109]. Moreover, differential methylation at loci such as ZFHX3 (cg07786668) and SMARCA4 (cg17218495) has been identified as independently associated with AMI, implicating epigenetic dysregulation of genes involved in transcriptional control and chromatin remodeling in the acute onset of AMI [110].

#### 2.4.2 Regulation by ncRNAs

ncRNAs, particularly miRNAs and ncRNAs, play pivotal regulatory roles in the pathophysiological processes of AMI [111]. Among them, highly conserved miRNAs are key modulators of cardiac and vascular morphology and function [112]. MicroRNA‐21 (miR‐21) is the most abundantly expressed miRNA in cardiac macrophages [113] and is significantly upregulated in patients with ACS [114]. In the setting of AMI, activation of the miR‐21/Hif‐1α axis in splenic marginal zone B cells (MZBs) promotes the secretion of CCL7, which in turn recruits Ly6C^High^ monocytes to infiltrate the myocardium, ultimately contributing to adverse cardiac remodeling after MI [33]. In addition, under diabetic conditions, I/R injury may be exacerbated through the *Fusobacterium nucleatum*–*Lactobacillus* oral–gut axis, potentially by upregulating miR‐21, thereby aggravating myocardial damage [115]. In addition to miR‐21, several miRNAs that are highly and specifically expressed in cardiomyocytes, such as miR‐1, miR‐208, and miR‐499, are significantly downregulated following AMI. This reduction may be associated with activation of the inflammatory response and structural damage in myocardial tissue [116].

lncRNAs are key regulators of cardiac development and disease. They can sponge miRNAs, thereby preventing miRNAs from binding to their target mRNAs and modulating gene expression [117]. During myocardial ischemia, downregulation of lncRNA H19 impairs the activation of autophagy, leading to reduced cellular clearance capacity and exacerbating ischemic injury [118]. During I/R, downregulation of lncRNA H19 also weakens its inhibitory effect on the miR‐877‐3p/Bcl‐2 axis, thereby promoting mitochondrial apoptosis and ultimately aggravating myocardial I/R injury [119]. In contrast, lncRNA Metastasis‐Associated Lung Adenocarcinoma Transcript 1 (MALAT1) is upregulated after MI. By targeting miR‐26b‐5p and upregulating Mfn1, MALAT1 helps restore mitochondrial dynamics and inhibit mitochondria‐dependent apoptosis, thus contributing to microvascular repair following MI [120]. Moreover, MALAT1, which is significantly upregulated after I/R, can also sponge miR‐30e, miR‐126, and miR‐155, thereby negatively regulating their respective targets CRP, HPSE, and EDN1, suggesting a broader role for MALAT1 in inflammatory regulation [121]. lncRNA SNHG15 is significantly upregulated on Day 7 after MI. It competitively binds to miR‐665, preventing miR‐665 from interacting with kinase insert domain receptor (KDR). This disinhibition activates VEGF signaling in endothelial cells, thereby enhancing their endogenous repair capacity in response to the pathophysiological remodeling following MI [122]. LncRNA 2810403D21Rik/AK007586/Mirf is significantly upregulated during MI. It contributes to ischemic myocardial injury by suppressing autophagy through the regulation of miR‐26a [123]. However, most current studies remain focused on validating individual regulatory axes, lacking comprehensive investigations into the spatiotemporal expression dynamics and interaction networks. Future research is needed to further explore their tissue‐specific functions and clinical translational potential.

#### 2.4.3 RNA Methylation

Eukaryotes possess over 100 different types of RNA modifications that modify mRNA and ncRNA, among which N6‐methyladenosine (m6A) is the most abundant type, where the nitrogen atom at the sixth position of adenosine undergoes methylation [124]. The dynamic balance of m6A is primarily regulated by methyltransferases, demethylases, and methyl‐binding proteins. Methyltransferases include METTL3, METTL14, WTAP, and RBM15; methyl‐binding proteins include YTHDF1‐3, YTHDC1‐3, and hnRNPs; while demethylases include ALKBH5 and FTO.

In the progression of AMI, the levels of m6A methylation are significantly elevated. Upregulated methyltransferases and methyl‐binding proteins, along with downregulated demethylases, contribute to myocardial injury or MIRI through various regulatory pathways including inflammation, oxidative stress, autophagy, apoptosis, and ferroptosis [125]. In hypoxic myocardial cells, the methyltransferase METTL3 modifies the m6A of the autophagy‐related gene ATG7 mRNA, promoting its degradation via recognition by YTHDF2 [126]. Under I/R conditions, METTL3 methylates the 3'‐UTR region of TFEB pre‐mRNA, promoting its interaction with heterogeneous nuclear ribonucleoprotein D (HNRNPD), leading to reduced TFEB expression, and jointly inhibiting protective autophagy [127]. Following MI, METTL14 methylates pri‐miR‐5099, promoting its maturation into miR‐5099‐3p, which in turn suppresses the expression of E74‐like ETS transcription factor 1 (ELF1), inducing myocardial cell pyroptosis [128]. METTL14 also regulates myocardial apoptosis through the Akt/mTOR signaling pathway after I/R [129]. Additionally, the expression of wilms tumor 1‐associated protein (WTAP) is downregulated during I/R, impairing its m6A modification of lncRNA Snhg1, which disrupts the miR‐361‐5p/ optic atrophy protein 1 (OPA1) axis responsible for mitochondrial fusion, thereby affecting myocardial mitochondrial homeostasis and cell survival [130]. In contrast, RNA binding motif protein 15 (RBM15) is significantly upregulated during MI and exerts a cardioprotective effect by stabilizing NEDD8 activating enzyme E1 subunit 1 (NAE1) mRNA, alleviating apoptosis [131]. Regarding methyl‐binding proteins, hnRNPA2B1 stabilizes PFN2 mRNA in an m6A‐dependent manner, which reduces ferritin heavy chain 1 (FTH1) expression and promotes myocardial ferroptosis, aggravating MIRI [132]. On the demethylation regulatory level, I/R induces a downregulation of FTO expression. Overexpression of FTO can mitigate oxidative stress and enhance mitochondrial biogenesis by demethylating PGC‐1α mRNA [133]. Furthermore, FTO overexpression inhibits the ubiquitination and subsequent degradation of β‐catenin mediated by Cbl proto‐oncogene (CBL) through demethylation of CBL mRNA, thereby suppressing NLRP3 inflammasome‐mediated pyroptosis, ultimately alleviating MIRI [134]. Conversely, overexpression of the demethylase ALKBH5 demethylates YTHDF1 mRNA, increasing YTHDF1 expression and promoting Fth1 protein translation, which in turn suppresses ferroptosis and alleviates MIRI [135]. With regard to specific therapeutic approaches, recent studies have shown that exercise training can attenuate myocardial injury by modulating the m6A methylation pathway, thereby providing new molecular targets and intervention strategies for the development of RNA methylation‐based protective therapies and rehabilitation methods [136, 137].

#### 2.4.4 Histone Modifications

Histone modifications are actively involved in regulating key pathological processes in AMI, including cardiomyocyte apoptosis, inflammation, and tissue repair. Common types of histone modifications include acetylation, methylation, phosphorylation, ubiquitination, and the more recently identified lactylation [138, 139]. In terms of acetylation, HDAC4 and HDAC5 are upregulated in cardiomyocytes during AMI, promoting apoptosis and exacerbating myocardial injury by suppressing the expression of anti‐apoptotic genes [140]. I/R also increases HDAC activity in cardiomyocytes, thereby promoting myocardial injury. Inhibition of HDACs may alleviate I/R‐induced damage by modulating the mitochondria‐dependent apoptotic pathway through Akt signaling [141]. In the context of histone methylation, the H3K27me3 demethylase JMJD3 is rapidly upregulated during the early stages of AMI and contributes to disease pathogenesis by activating inflammatory genes such as IL‐1β [142, 143]. In contrast, the H3K9me2 demethylase KDM3A exerts cardioprotective effects during I/R by activating the PI3K/Akt signaling pathway and attenuating pyroptosis in cardiac microvascular endothelial cells (CMECs) [144]. Regarding phosphorylation, I/R triggers the phosphorylation of the histone variant H2AX, which, while involved in DNA repair, can also accumulate and activate the p53/JNK pathway, leading to mitochondrial damage and cardiomyocyte apoptosis [145]. Ubiquitination is also critically important. The ubiquitin‐proteasome system (UPS) plays a central role in protein quality control. Within this system, the E3 ubiquitin ligase TRIM21 exacerbates post‐MI cardiac injury by promoting M1 macrophage polarization and enhancing the inflammatory response through activation of the PI3K/Akt pathway [146]. TRIM6 aggravates MIRI by promoting cardiomyocyte apoptosis via activation of the IKKε/STAT1 signaling pathway [147]. In addition, USP7 has been found to be upregulated during MIRI. Targeting USP7‐mediated degradation of Keap1 may represent a potential mechanism for reducing myocardial I/R injury [148]. Emerging modifications such as histone lactylation have also garnered increasing attention. Histone lactylation promotes the early and remote activation of monocyte‐derived reparative genes following MI, offering a novel direction for maintaining immune homeostasis and facilitating cardiac repair after MI [149].

#### 2.4.5 Ventricular Remodeling

**FIGURE 3:**
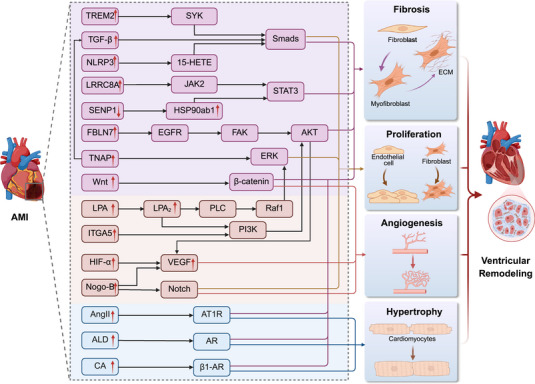
Key molecular mechanisms underlying ventricular remodeling after AMI. After AMI, coordinated signaling pathways regulate fibrosis, cell proliferation, angiogenesis, and cardiomyocyte hypertrophy, collectively driving structural and functional changes in the ventricle. AMI, acute myocardial infarction; ALD, aldosterone; Ang II, angiotensin II; AR, aldosterone receptor; AT1R, angiotensin II type I receptor; β1‐AR, β1‐adrenergic receptor; CA, catecholamine; ECM, extracellular matrix; EGFR, epidermal growth factor receptor; ERK, extracellular signal‐regulated kinase; FBLN7, fibulin 7; HIF‐α, hypoxia‐inducible factor 1‐α; ITGA5, integrin α5; LPA, lysophosphatidic acid; LPA_2_, lysophosphatidic acid receptor 2; TNAP1, tissue nonspecific alkaline phosphatase; TREM2, triggering receptor expressed on myeloid cells 2; VEGF, vascular endothelial growth factor.

Ventricular remodeling after AMI is a progressive change in the structure, size, and function of the heart following infarction and is a critical process leading to heart failure after myocardial infarction. Pathologically, it is characterized by cardiomyocyte hypertrophy, extensive myocardial fibrosis, and abnormal cell proliferation. Following AMI, the process of ventricular remodeling begins with cardiomyocyte death, accompanied by inflammation, fibrosis, angiogenesis activation, and sustained multiple drivers from the neuroendocrine system (Figure 3). During the acute phase, neutrophils and M1 macrophages infiltrate in large numbers, secreting TNF‐α, IL‐1β, MMPs, and other factors, leading to extracellular matrix (ECM) degradation [150], which may cause an expansion of the infarction area. Subsequently, neutrophils are replaced by macrophages, and their phenotype shifts from M1 to the anti‐inflammatory/reparative M2 type. M2 macrophages secrete anti‐inflammatory factors such as TGF‐β, which is a key driver initiating reparative remodeling. The TGF‐β/Smads and Wnt/β‐catenin signaling pathways promote the differentiation of fibroblasts into myofibroblasts. Myofibroblasts lead to substantial ECM deposition and synthesis of type I and II collagen, which are major pathways promoting myocardial fibrosis and altering ventricular morphology [151, 152].

In addition to the classic pathways mentioned, various molecular mechanisms also regulate fibroblast activation following AMI. The NLRP3 inflammasome contributes to fibroblast transformation through the 15‐HETE/Smad pathway [153], LRRC8A activates the downstream JAK2‐STAT3 signaling pathway [154], and Fibulin7 (FBLN7) participates in the process through the EGFR/FAK/AKT signaling pathway [155]. Sentrin‐specific protease 1 (SENP1) has been found to be downregulated after MI, leading to increased ubiquitination of HSP90ab1, which, along with STAT3 activation and Fn secretion, accelerates fibrosis [156]. In the later stages, gelsolin drives the differentiation of the TREM2⁺Spp1⁺ macrophage subpopulation, and their accumulation further increases the crosstalk with fibroblasts, exacerbating pathological ventricular remodeling [157]. Fibroblast proliferation is also an important mechanism in the progression of cardiac fibrosis. Tissue nonspecific alkaline phosphatase (TNAP) not only activates fibroblasts through the TGF‐β/Smads pathway but also promotes fibroblast proliferation by activating the ERK1/2 signaling pathway [158]. The transmembrane innate immune receptor triggering receptor expressed on myeloid cells 2 (TREM2) is highly expressed in macrophages after MI. It inhibits the transcription of mitochondrial NAD^+^ transporter SLC25A53 via the SYK‐Smad4 pathway, promotes the generation of immunometabolic product kynurenine, and thus inhibits cardiomyocyte apoptosis and enhances fibroblast proliferation, significantly improving cardiac repair [159].

Ventricular remodeling after MI also involves angiogenesis, primarily occurring in the infarcted border zone and extending toward the infarct core. Angiogenesis involves the sprouting of new vessels from existing endothelial cells. Myocardial hypoxia induces the upregulation of hypoxia‐inducible factor‐1 α (HIF‐1α) and its key target gene vascular endothelial growth factor (VEGF), which is a core driver of angiogenesis [160]. The Wnt/β‐catenin pathway also plays an important role in angiogenesis [161]. Moreover, MI upregulates macrophage‐specific integrin α5 (ITGA5) expression, which promotes VEGF expression and facilitates angiogenesis through the PI3K/Akt and FAK pathways [162]. Nogo‐B promotes angiogenesis after MI by activating endothelial and cardiac Notch signaling pathways, leading to endothelial cell proliferation [163]. Lysophosphatidic acid receptor 2 (LPA_2_) in endothelial cells is also an important factor in angiogenesis. Research shows that LPA_2_ promotes endothelial cell proliferation and maintains vascular homeostasis through the PI3K‐Akt/PLC‐Raf1‐Erk pathway [164].

Regarding the neuroendocrine system, after MI, renin, angiotensin, angiotensin II (Ang II) type I receptor (AT1R), and aldosterone are significantly increased in the infarcted myocardium. Ang II activates the AT1R in cardiomyocytes and fibroblasts, causing cell hypertrophy and fibrosis [165]. Aldosterone also promotes the synthesis of collagen and ECM deposition by fibroblasts, further exacerbating fibrosis [166]. Additionally, the sympathetic nervous system is activated after MI, with excessive catecholamine (CA) release activating β1‐adrenergic receptors (β1‐AR), leading to cardiomyocyte hypertrophy and myocardial fibrosis. With the progress of spatial omics, it has been found that ventricular remodeling extends beyond the infarct zone to the peri‐infarct area and distant myocardium, involving the spatial–temporal activation of the immune–fibroblast axis and metabolic reprogramming, driving overall cardiac repair [167]. This provides a multi‐dimensional perspective for future mechanistic exploration of AMI and potential new therapeutic targets for treatment.

## 3 Mechanisms of Inter‐Organ Crosstalk in AMI

**FIGURE 4:**
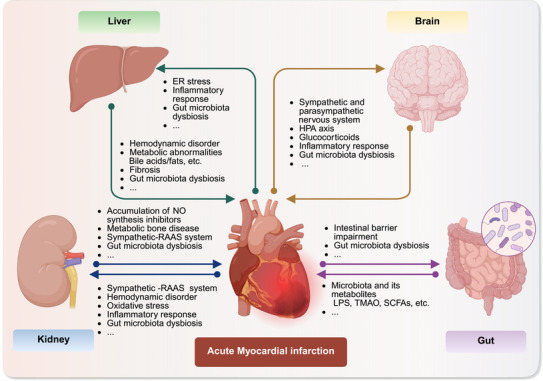
Multiorgan crosstalk in AMI pathophysiology. AMI can induce organ‐specific pathological changes such as liver inflammation, renal hemodynamic load, activation of the neuroendocrine system in the brain, and intestinal barrier disruption. These changes, in turn, exacerbate cardiac injury through mechanisms such as enhanced inflammation, oxidative stress, and metabolic dysregulation, thereby establishing a vicious cycle between the heart and multiple organs. AMI, acute myocardial infarction; ER, endoplasmic reticulum; HPA, hypothalamic–pituitary–adrenal; LPS, lipopolysaccharide; RAAS, renin‐angiotensin‐aldosterone system; SCFA, short‐chain fatty acids; TMAO, trimethylamine N‐oxide.

AMI can trigger complex inter‐organ crosstalk, significantly affecting the prognosis of patients. Following AMI, the heart's pumping capacity is markedly diminished, resulting in systemic organ hypoperfusion, which serves as a critical driver of damage to other organs. In addition, the coexistence of cerebral infarction and MI, brain–heart interactions, the liver's role in addressing myocardial energy crisis via lipid metabolism reprogramming, systemic regulation of the sympathetic and parasympathetic nervous systems, and inter‐organ interaction mechanisms underlying pan‐vessel disease underscore that myocardial infarction is fundamentally a systemic disease characterized by multi‐organ crosstalk (Figure 4) [168, 169]. This section will focus on elucidating the specific molecular mechanisms underlying multi‐organ interactions to inform AMI treatment strategies. Consequently, the shared risk factors of the aforementioned diseases are beyond the primary scope of this discussion.

### 3.1 Heart–Gut Crosstalk

The intestine, as the largest immune organ and a major microbial habitat in the human body, plays a critical role in systemic immune regulation and the function of distant organs through the maintenance of barrier integrity and microbiota homeostasis. Following AMI, the abrupt decline in cardiac output combined with excessive sympathetic activation leads to sustained intestinal hypoperfusion. This, along with ischemia‐induced gut dysbiosis, synergistically compromises the intestinal barrier, ultimately resulting in abnormal translocation of gut microbiota and their metabolites [170]. Gram‐negative bacterial LPS translocates from the intestinal lumen into the circulation, where it activates the TLR4/MyD88/NF‐κB signaling pathway, triggering massive release of pro‐inflammatory cytokines such as TNF‐α and IL‐1β. This initiates an inflammatory cascade and promotes cathepsin G release, accelerating thrombosis and exacerbating tissue injury [171]. Glucagon‐like peptide‐2 (GLP‐2) analogs have been shown to confer cardioprotective effects during MIRI by attenuating gut microbiota translocation and inflammation [170]. Preventive I/R interventions have also been reported to increase γ‐aminobutyric acid (GABA) levels, thereby suppressing macrophage NLRP3 inflammasome activation and IL‐1β release, ultimately providing cardioprotection after I/R [172].

AMI is associated with marked alterations in gut microbiota composition. Eight weeks after AMI, overall gut microbial abundance decreases. At the phylum level, the relative abundance of *Firmicutes* is reduced, while *Bacteroidota* is increased. At the genus level, potentially harmful taxa such as *Lachnospiraceae_UCG‐001* are enriched, whereas beneficial genera including *Alistipes*, *Ruminococcus*, *Allobaculum*, and *Oscillospiraceae_UCG‐005* are diminished [173]. Gut microbial metabolic profiles also shift substantially post‐AMI. Levels of several metabolites, such as trimethylamine N‐oxide (TMAO), methionine, deoxycholic acid (DCA), phenylacetylglutamine (PAGln), and indoxyl sulfate (IS), are elevated and have been strongly associated with poor outcomes [174, 175]. Conversely, beneficial bacteria and metabolites decline, notably *Lactobacillus* and *short‐chain fatty acids* (SCFAs), particularly acetate, butyrate, and propionate. Supplementation with *Lactobacillus* or SCFAs after MI can modulate immune cell ratios, promote cardiac repair, and improve cardiac function. Additionally, *Lactobacillus* has been reported to activate the Sirt1/Nrf2/HO‐1 pathway, thereby suppressing inflammation, oxidative stress, ferroptosis, and apoptosis, protecting against myocardial I/R injury [176]. Interestingly, some beneficial bacteria increase after AMI. Butyrate‐producing species such as *Butyricimonas virosa* and *Streptococcus parasanguinis* are enriched, leading to elevated β‐hydroxybutyrate levels, which contribute to post‐MI cardiac repair [177].

From a therapeutic perspective, ethanol extract of *Pueraria lobata* (EEPL) has been shown to alleviate myocardial injury after AMI by modulating gut microbiota dysbiosis, particularly *Lachnoclostridium*, and increasing bile acid levels [178]. Beyond its influence on AMI, the gut microbiota also mediates bidirectional communication between the intestine and distant organs such as the brain, liver, and kidney in the post‐AMI setting. Targeting the maintenance of intestinal barrier function and microbiota homeostasis may represent a promising strategy to interrupt the vicious cycle of multi‐organ cross‐talk after AMI.

### 3.2 Heart–Brain Crosstalk

Brain injury and acute ischemic stroke (AIS) can induce or exacerbate AMI, while AMI can also exert adverse effects on brain tissue and function, thereby further aggravating cardiac damage. The underlying mechanisms involved include inflammatory responses, activation of the hypothalamic–pituitary–adrenal (HPA) axis, dysregulation of the sympathetic and parasympathetic nervous systems, as well as alterations in the gut microbiota. Following MI, the activation of cardiac mechanoreceptors triggers central sympathetic excitation via vagal afferent fibers, resulting in cerebral hypoperfusion and stress‐induced neuronal injury, while enhanced sympathetic output exacerbates myocardial remodeling. Activation of the HPA axis results in a marked increase in corticosterone levels, which is especially prominent under stress and adversely impacts cardiac function [179, 180]. Prolonged cortisol elevation exhibits potential neurotoxic effects, further impairing neuronal activity and the blood–brain barrier (BBB), thereby forming a positive feedback loop of heart–brain injury [181]. The paraventricular nucleus directly projects to the rostral ventrolateral medulla (RVLM) of the medulla oblongata, facilitating the integration of cardiac afferent signals, baroreceptor activity, and inputs from higher brain regions with sympathetic nerve output to the heart [182, 183]. The activated sympathetic nervous can aggravate left ventricular remodeling after myocardial infarction. Furthermore, the brain exerts a central regulatory effect on the autonomic nervous system [184]. Dysfunction of the autonomic nervous system not only independently predicts adverse outcomes in AIS patients [185] but also promotes myocardial injury [186, 187], serving as a critical common mechanism underlying multi‐organ interactions [188, 189].

MI may also contribute to the disruption of the BBB [190, 191], facilitating the infiltration of neutrophils and peripheral macrophages into ischemic brain tissue. Brain‐derived antigens and extracellular vesicles can traverse the compromised BBB and interact with peripheral immune cells [192], thereby establishing a heart–brain interaction. During this process, immune‐inflammatory responses are also triggered. Following AMI or AIS, local immune‐inflammatory responses can rapidly evolve into systemic inflammation, releasing cytokines such as IL‐1β, IL‐6, and TNF‐α, which may contribute to secondary myocardial injury [193, 194]. These targets are also key areas for the development of anti‐inflammatory drugs aimed at reducing neuroinflammation and cardiac remodeling to achieve dual benefits. Interventions targeting glial cells, TLRs pathways, or mitochondrial function may concurrently mitigate the post‐ischemic pathological processes in both the brain and heart [195]. Ginsenoside Rb1 reduces ROS generation by inhibiting mitochondrial complex I, thereby suppressing excessive activation of astrocytes [196], suggesting that targeting astrocytes may represent a potential strategy for cardioprotection and neuroprotection [197, 198].

In addition to local ischemic preconditioning in the heart and brain, remote ischemic preconditioning (RIPC) represents a promising therapeutic strategy for mitigating the adverse effects of myocardial infarction and stroke on cardiac function [199, 200, 201, 202, 203]. This process involves multiple mechanisms, including neuro‐humoral regulation, oxidative stress modulation, and extracellular vesicle‐mediated signaling [202, 204, 205]. Based on a similar mechanism, RIPC can synergistically inhibit inflammation and improve metabolism, enhancing the protective effect on the heart and brain. The gut microbiota serves as a critical distal regulatory component. MI can induce intestinal barrier dysfunction, leading to gut microbiota dysbiosis (e.g., overgrowth of Proteobacteria) and translocation. Subsequently, bacteria and their metabolites (such as LPS) enter the circulatory system, triggering systemic inflammation and thereby promoting heart–brain crosstalk [170, 206]. Gut microbiota‐derived metabolites, such as TMAO, promote atherosclerosis, platelet aggregation, and myocardial fibrosis by activating the NF‐κB signaling pathway and the NLRP3 inflammasome. Simultaneously, these effects exacerbate neuroinflammation and blood–brain barrier dysfunction [207, 208, 209].

In addition to pathophysiological mechanisms, the functions of the central autonomic nervous system, such as arousal and sleep regulation [210, 211] and emotional modulation [212, 213], not only predict the risk and prognosis of MI but also represent critical therapeutic targets. Among these, signaling pathways such as TNF and FOXO may serve as key mediators of organ crosstalk [210, 211]. Inhibition of FOXO activity has been shown to significantly reduce cardiomyocyte apoptosis and enhance survival [214]. In summary, the heart–brain interaction establishes a pathological network via multi‐dimensional mechanisms. Targeting key pathways or employing RIPC and other strategies offers promising avenues for blocking damage progression and achieving multi‐organ protection.

### 3.3 Heart–Kidney Crosstalk

The heart–kidney interaction mechanism encompasses hemodynamic disturbances, neuroendocrine activation, immune‐inflammatory responses, and intercellular communication. Beyond the classical etiologies of cardiorenal syndrome, myocardial necrosis following MI releases DAMPs and enhances oxidative stress. This process can activate the renal TLR4/NF‐κB pathway, thereby inducing inflammation and apoptosis in renal tubular epithelial cells, while simultaneously elevating systemic levels of IL‐6 and TNF‐α [215, 216]. Xin‐Ji‐Er‐Kang mitigates renal injury by inhibiting oxidative stress via the Nrf2/HO‐1 pathway [217]. Renal I/R injury can activate the NLRP3 inflammasome, which inhibits myocardial contractility and promotes myocardial fibrosis through inflammatory factors and downstream NF‐κB [218, 219]. In terms of treatment, the recently identified myokine Irisin can mitigate vascular smooth muscle cell (VSMC) calcification in chronic kidney disease (CKD) conditions and decrease cardiovascular risk by inducing autophagy and suppressing the NLRP3 inflammasome [220].

In addition, intestinal dysbiosis promotes bidirectional heart–kidney damage via metabolic toxins, activation of innate immunity, and neurohumoral mechanisms. Analogous to the heart–brain axis, microbial metabolites can induce injury to both the cardiovascular system and kidneys [221]. In CKD, the accumulation of nitric oxide (NO) synthase inhibitors, abnormal metabolic bone disease markers, and activation of the sympathetic and renin‐angiotensin‐aldosterone system (RAAS) further exacerbate endothelial dysfunction and ventricular remodeling [222, 223, 224]. Notably, myocardial ischemia can induce renal injury via inflammatory mechanisms [225, 226], while venous congestion resulting from heart failure can further exacerbate kidney lesions in a reciprocal manner [215]. Ultimately, these processes accelerate the comorbidity of heart and kidney diseases through inflammatory and fibrotic pathways [227]. Currently, the multi‐target mechanisms of GLP‐1RA and SGLT2 inhibitors have garnered significant attention. Their combination can synergistically enhance cardiac and renal outcomes [228, 229]. Nevertheless, as these drugs exert cardioprotective effects via indirect mechanisms, further investigation into their application in patients with myocardial infarction and cardiorenal syndrome is warranted.

### 3.4 Heart–Liver Crosstalk

AMI is likely to induce acute heart failure, which in turn can trigger acute or chronic cardiorenal syndrome. Associated with hepatic congestion, this condition may impair bile acid excretion, further exacerbating myocardial dysfunction [230]. On the other hand, in patients with chronic liver disease, the accumulation of bile acids or lipids may adversely affect myocardial function, thereby establishing a vicious cycle. Metabolic dysfunction‐associated fatty liver disease (MAFLD) are independent risk factors for MI and stroke, increasing the risk of fatal/non‐fatal cardiovascular events by 45% [231]. Metabolic disturbances and severe hepatic fibrosis are associated with a further elevation in cardiovascular risk among patients with MIAFLD [232]. The liver serves as the primary organ for bile acid and PCSK9 production. The farnesoid X receptor (FXR) agonist GW4064 can activate the FXR/AMPK/PGC1α pathway in adipocytes, thereby remotely enhancing cardiac remodeling following MI [233]. PCSK9 promotes platelet activation and in vivo thrombosis through binding to platelet CD36, thereby increasing the risk of MI [230, 234]. Similarly, the liver can produce bone morphogenetic protein 9 (BMP9). BMP9 alleviates myocardial infarction injury by improving lymphatic drainage and enhancing the mitochondrial β‐oxidation rate‐limiting enzyme 2,4‐dienoyl‐CoA reductase 1 (DECR1) [235].

Myocardial I/R induces the release of small sEVs, which target and inhibit endoplasmic reticulum degradation enhancing alpha‐mannosidase‐like protein 3 (EDEM3). This inhibition subsequently activates the PERK‐CHOP/ATF6‐EDEM endoplasmic reticulum (ER) stress pathway, leading to systemic metabolic dysregulation [236]. The traditional view suggests that the cardioprotective effects of spironolactone are primarily mediated through direct actions on the cardiac mineralocorticoid receptor (MR). However, the heart–liver axis may also play a critical role. Following MI, plasma IL‐6/STAT3 levels rapidly increase, which in turn regulates the liver MR/fibroblast growth factor 21 (FGF21) axis and further influences cardiac injury. Therefore, targeting hepatic FGF21 with agonists could potentially reduce heart failure post‐MI [237].

## 4 Diagnosis

**FIGURE 5:**
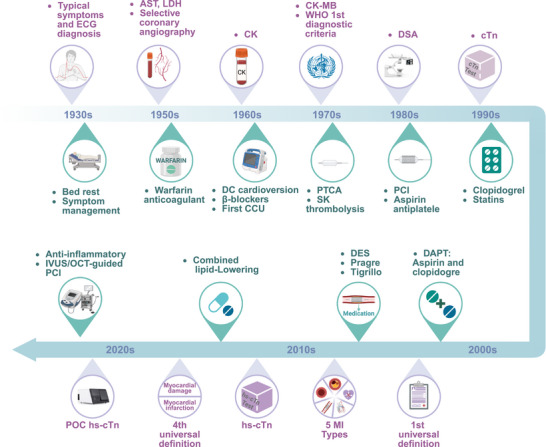
Evolution of diagnostic criteria and clinical management in AMI. The diagnostic criteria for AMI have progressively advanced from reliance on symptoms and ECG to the integration of troponin detection technology, with diagnostic classification becoming increasingly precise. Clinical management has transitioned from basic supportive care to the systematic adoption of comprehensive drug regimens and routine reperfusion therapy. AMI, acute myocardial infarction; AST, aspartate aminotransferase; CK, creatine kinase; CK‐MB, creatine kinase MB; DAPT, dual antiplatelet therapy; DSA, digital subtraction angiography; DES, drug‐eluting stent; hs‐cTn, high‐sensitivity cardiac troponin; IVUS, intravascular ultrasound; LDH, lactatedehydrogenase; OCT, optical coherence tomography; PCI, percutaneous coronary intervention; POC, point‐of‐care; PTCA, percutaneous transluminal coronary angioplasty; SK, streptokinase.

The evolution of AMI diagnostic criteria has spanned a century (Figure 5), progressing from initial reliance on symptom assessment alone to the integration of electrocardiography and cardiac enzyme testing. Today, widespread coronary intervention has enabled visual diagnosis, while the application of multimodal imaging has achieved comprehensive evaluation. In recent years, the continuous refinement of AMI classification systems, the widespread adoption of point‐of‐care (POC) testing, the discovery of multi‐omics biomarkers, and the rapid advancement of AI tools are collectively propelling current diagnostic capabilities toward a new phase characterized by speed, precision, and non‐invasiveness.

### 4.1 Evolution of Diagnostic Criteria

In the 1930s and 1940s, the diagnosis of AMI primarily relied on the presence of typical clinical symptoms and their direct association with ST‐segment elevation on ECGs [238]. By the 1950s, serum biomarkers such as creatine kinase (CK), lactate dehydrogenase (LDH), and aspartate aminotransferase (AST) were introduced into the diagnostic workflow, although their sensitivity and specificity were significantly limited [239]. In the 1970s, the introduction of CK‐MB, a biomarker with greater cardiac specificity, led to the World Health Organization's (WHO) first formal diagnostic criteria for MI, based on the triad of clinical symptoms, electrocardiographic changes, and cardiac enzyme levels. This initial MI diagnostic standard evolved into the 3:2 model, requiring at least two of the following three: history of chest pain, characteristic ECG changes, and dynamic alterations in cardiac enzymes, with further classification into non‐transmural MI or subendocardial MI and transmural MI according to Q‐wave morphology on ECG [240, 241]. Subsequently, the discovery of highly sensitive biomarkers such as cardiac troponins (cTn) and myoglobin (MyO) established cardiac enzyme testing as a cornerstone in the diagnosis of AMI [242]. Meanwhile, breakthroughs in imaging technologies further advanced the diagnostic framework for AMI. In 1958, the first selective coronary angiography via the brachial artery marked the beginning of coronary angiographic techniques [243]. This was followed by the widespread adoption of transfemoral access (TFA) and transradial access (TRA) approaches for coronary angiography [244, 245]. The development of digital subtraction angiography (DSA) in the 1980s further provided a critical tool for the visualization of coronary arteries, enhancing the diagnostic precision for AMI [246].

Since the beginning of the 21st century, the integration of high‐sensitivity cardiac biomarkers and advanced imaging technologies has driven significant improvements in the precision of AMI diagnosis. In 2000, the first universal definition of MI was published, defining MI as “myocardial cell death resulting from ischemia.” According to this definition, diagnosis requires a significant elevation in cardiac biomarkers such as CK‐MB or troponin, in combination with clinical evidence of myocardial ischemia, which includes ischemic symptoms, electrocardiographic changes, angiographic confirmation, or pathological findings [247]. The second universal definition of MI, published in 2007, formally established cTn as the diagnostic gold standard and, for the first time, categorized MI into five types based on distinct pathophysiological mechanisms and clinical contexts, moving beyond traditional classification schemes [248]. In 2017, the International Classification of Diseases (ICD) introduced a new diagnostic code specifically for type 2 MI [249] The fourth universal definition, released in 2018, further refined the criteria to distinguish myocardial injury from infarction, expanded the MI classification to include myocardial infarction with non‐obstructive coronary arteries (MINOCA) and Takotsubo syndrome, and emphasized the importance of dynamic monitoring of high‐sensitivity troponin (hs‐cTn) as well as the integrated application of imaging techniques, such as cardiac magnetic resonance (CMR) and coronary computed tomography (CT) [250]. Based on continuously refined diagnostic strategies, the current diagnosis of AMI has achieved comprehensive integration of clinical symptoms, electrocardiography, high‐sensitivity biomarkers, and multimodality imaging. The 2025 ACC/AHA/ACEP/NAEMSP/SCAI guidelines specify that the diagnosis of AMI should be established through an integrated assessment of typical or atypical ischemic symptoms, characteristic electrocardiographic changes, and elevations with dynamic changes in high‐sensitivity cardiac troponin (hs‐cTn), supplemented by imaging modalities to determine the extent and nature of the lesion [251].

### 4.2 Current Progress and Challenges

#### 4.2.1 Limitations of the Existing Diagnostic Classification

Despite the increasingly refined classification of AMI, the widely adopted universal five‐type classification system has gained broad recognition in both academia and clinical practice, yet it still faces considerable challenges in real‐world application. Approximately 5% of patients diagnosed with type 2 MI are actually misclassified cases of type 1 or type 4b MI. Moreover, type 2 MI encompasses both coronary and non‐coronary mechanisms, leading to therapeutic ambiguity. For type 3 MI, the lack of biochemical and imaging evidence makes it difficult to identify the underlying mechanism, thereby limiting its value in guiding clinical decision‐making [9, 252]. In response to these limitations, some scholars have proposed a novel classification framework centered on clinical features, which categorizes MI into three groups: spontaneous MI (driven by coronary pathology), secondary MI (caused by oxygen supply–demand imbalance due to acute illness), and procedural MI (associated with complications of revascularization procedures). This framework emphasizes the role of imaging‐based evidence in enhancing diagnostic specificity and may help address the diagnostic heterogeneity observed in both clinical practice and research settings [9]. However, whether this new classification can more accurately guide diagnosis, optimize individualized treatment strategies, and improve patient outcomes requires extensive validation and evaluation in large‐scale, real‐world clinical practice.

#### 4.2.2 Advances in Diagnostic Methods

Current advances in AMI diagnostics are shifting toward faster, more precise, and less invasive approaches. Point‐of care (POC) testing for high‐sensitivity cardiac troponin (hs‐cTnI) enables rapid single or serial measurements, significantly shortening the diagnostic window. This approach allows for the safe rule‐out of low‐risk patients and the accurate identification of high‐risk individuals [11, 253, 254]. Luca Koechlin et al. demonstrated that a novel single‐use immunofluorescence POC device (hs‐cTnI‐SPINCHIP), utilizing a 0/1‐h algorithm with dynamic thresholding, can further improve diagnostic sensitivity. Its performance was shown to be comparable to that of validated central laboratory assays [255]. In the field of imaging, a novel deep learning approach based on cine‐generated enhancement (CGE) has been developed, which can generate images comparable to those obtained by late gadolinium enhancement (LGE) using only standard cine cardiac magnetic resonance (cine CMR) images. This technique offers a promising non‐invasive alternative for myocardial tissue characterization [10]. Furthermore, multi‐omics technologies integrating genomics, proteomics, metabolomics, and epigenomics have revealed the molecular regulatory networks involved in the early stages of AMI and identified multiple potential novel biomarkers, such as gut microbiota‐derived metabolites (e.g., TMAO) and circulating miRNAs (e.g., miR‐1, miR‐499), providing new perspectives for early AMI detection [106, 116, 256, 257]. These technological advancements are expected to drive the development of a multi‐modal, integrative diagnostic paradigm for AMI, with significant potential to optimize healthcare resource allocation, reduce unnecessary testing and hospitalizations, and improve clinical outcomes.

#### 4.2.3 Applications of AI

In addition, the introduction of AI‐based diagnostic tools has further enhanced the diagnostic performance for AMI. Clinical decision support systems such as the Collaboration for the Diagnosis and Evaluation of Acute Coronary Syndrome (CoDE‐ACS) and the myocardial‐ischemic‐injury‐index (MI^3^), developed using machine learning algorithms that integrate hs‐cTnI levels with clinical features, have been shown to significantly improve diagnostic accuracy, compared to traditional approaches [258, 259]. Another machine learning based algorithm, Artificial Intelligence in Suspected Myocardial Infarction Study (ARTEMIS)‐POC, which integrates a single POC hs‐cTnI measurement, has also been shown to rapidly and safely rule out MI [11]. In addition, AI‐enhanced electrocardiography (AI‐ECG) has demonstrated superior diagnostic performance in identifying AMI, significantly outperforming traditional risk stratification tools such as the GRACE 2.0 score and high‐sensitivity troponin levels [260]. In risk prediction, AI‐based quantitative coronary plaque volume measurement and plaque burden assessment have achieved high‐precision prediction of AMI risk [261, 262]. The RETFound model, through analysis of retinal microvascular and neural structural changes, can effectively predict the risk of AMI within 3 years [263]. These AI‐driven methods address key limitations of traditional approaches, such as restricted applicability during early symptom onset and insufficient generalizability. Nevertheless, their external validity still requires confirmation in large prospective cohorts. Looking ahead, with continued technological advancements, the development of a multimodal, integrated AMI prevention and management model holds promise for improving early warning, precision prevention, and timely intervention, with significant potential to optimize healthcare resource allocation, reduce unnecessary testing and hospitalization, and improve clinical outcomes.

## 5 Clinical Management

The clinical management model for AMI has undergone a significant transformation. On one hand, a large body of high‐level evidence‐based research has laid the foundation for standardized drug treatment regimens. On the other hand, the emergence and widespread application of coronary revascularization techniques have achieved a milestone therapeutic breakthrough. Current research is focused on precision medicine, striving to continuously optimize treatment protocols and refine beneficiary populations. Concurrently, emerging strategies such as anti‐inflammatory therapy, myocardial cell regeneration, and gene editing are continually expanding new avenues for AMI treatment. Traditional Chinese medicine (TCM) has also demonstrated unique value in treatment. These measures collectively offer new hope for further reducing myocardial injury, promoting repair, and improving prognosis.

### 5.1 Evolution of Clinical Management

Since the 19th century, when nitroglycerin was first used to relieve angina pectoris, the clinical management of AMI had, for decades, primarily focused on bed rest and symptomatic relief (Figure 5). A paradigm shift in therapeutic strategies emerged in the 1960s, when the introduction of β‐blockers, which were proven to reduce myocardial oxygen demand and suppress malignant arrhythmias, together with the adoption of direct current (DC) cardioversion and the establishment of the first coronary care units (CCUs), markedly reduced AMI mortality associated with severe arrhythmias [264, 265, 266]. Since the 1970s, significant breakthroughs have been achieved in the field of antiplatelet therapy. Aspirin was established as a cornerstone treatment through the landmark ISIS‐2 trial [267]. The subsequent introduction of P2Y12 inhibitors, including clopidogrel, followed by prasugrel and ticagrelor, further optimized dual antiplatelet therapy (DAPT) regimens [268, 269, 270]. In 1994, statin therapy ushered in the era of lipid management with the publication of the 4S trial [271]. Subsequent studies such as IMPROVE‐IT and ODYSSEY further advanced the field by supporting the development of combination lipid‐lowering strategies [272, 273].

The evolution of coronary revascularization techniques has fundamentally reshaped the treatment paradigm for AMI. While the introduction of streptokinase thrombolysis in 1979 reduced AMI mortality by 30%, its limitations, including a low recanalization rate and high bleeding risk, spurred the development of more effective revascularization strategies [274]. The first percutaneous transluminal coronary angioplasty (PTCA) procedure was performed in 1977 [275], and it was first applied to AMI patients in 1983 [276]. In 1993, the first RCT demonstrated that direct PCI outperformed intravenous thrombolysis, solidifying PCI's role and marking the beginning of the reperfusion era [277]. Since then, PCI technology has advanced rapidly, with milestones including the clinical application of self‐expanding stents in 1987, the introduction and widespread adoption of balloon‐expandable stents and drug‐eluting stents (DES), as well as subsequent iterations of DES, which have significantly reduced the risk of restenosis [278]. In 2003, the ESC guidelines designated PCI as the preferred strategy for STEMI [279]. Alongside the evolution of reperfusion techniques, the concept of time windows has also progressed, with established standards such as the myocardial infarction interventional window and the time from first medical contact (FMC) to first device use driving the global establishment of chest pain center systems. Additionally, cardiac rehabilitation, which originated in the 1960s [280], has evolved from simple exercise‐based programs into comprehensive management models, proven to effectively reduce long‐term mortality. Currently, cardiac rehabilitation is recognized as an essential component of post‐discharge management for myocardial infarction and is included in clinical guidelines [251]. With these advances, mainstream treatment strategies for STEMI and NSTEMI have now been well established [251]. For NSTEMI, management is guided by risk stratification. High‐risk patients are prioritized for invasive strategies such as coronary angiography within 24 h, whereas patients at intermediate or low risk may be managed conservatively. Pharmacologic therapy is based on DAPT, anticoagulation, and intensive lipid‐lowering treatment, with revascularization performed when clinically indicated. In STEMI, the urgency of reperfusion therapy is emphasized. Primary percutaneous coronary intervention (PPCI) is the preferred strategy, with the recommended door‐to‐balloon time not exceeding 90 min. When timely PCI is not feasible, fibrinolytic therapy is administered. The pharmacologic regimen for STEMI is broadly similar to that of NSTEMI.

### 5.2 Current Progress and Challenges

#### 5.2.1 Extent of Revascularization Intervention

In the revascularization treatment of AMI, the PCI strategy continues to evolve toward optimization. The COMPLETE study demonstrated that, in patients with multivessel disease and STEMI, complete revascularization reduced the risk of cardiovascular death or new myocardial infarction by 26%, compared to PCI targeting only the culprit lesion after a median follow‐up of 3 years [281]. The optical coherence tomography (OCT) subgroup analysis revealed that 50% of STEMI patients exhibited vulnerable plaque morphology in non‐culprit vessels, with a higher prevalence of obstructive lesions, providing pathological support for the complete revascularization strategy in STEMI patients with multivessel disease [282]. However, the recent FULL REVASC trial found that, after a median follow‐up of 4.8 years, fractional flow reserve (FFR)‐guided complete revascularization did not reduce the risk of composite endpoints, including death, myocardial infarction, or revascularization [283]. Thus, the specific criteria for non‐culprit vessel intervention in STEMI patients remain controversial. Additionally, as the population ages, the proportion of elderly AMI patients is increasing. Elderly patients often present with unique clinical characteristics and comorbidity burdens, necessitating more tailored treatment strategies. Recent studies have increasingly focused on this special population. The FIRE study indicated that, in elderly MI patients aged over 75 years with multivessel coronary artery disease, complete revascularization guided by coronary physiology assessment significantly reduced ischemic events, compared to treating only the infarct‐related artery [284]. This finding was later corroborated by another meta‐analysis [285]. Conversely, in an RCT involving frail elderly patients with NSTEMI, the conventional invasive strategy showed no benefit during the days alive and out of the hospital (DAOH) period [286]. Therefore, further evidence is required to clarify the safety and efficacy of complete revascularization in elderly patients with myocardial infarction. Moreover, the clinical value of complete revascularization in NSTEMI patients remains uncertain, and we must await the results of ongoing large‐scale studies for guidance.

#### 5.2.2 Optimization of Antiplatelet Therapy

With the widespread adoption of next‐generation DES and potent P2Y12 receptor antagonists, the antiplatelet strategy for AMI patients post‐PCI has evolved from standardization toward individualized and precision‐based approaches. The primary goal is to balance ischemic and bleeding risks while maximizing clinical net benefit. In recent years, several large‐scale RCTs have provided robust evidence supporting the down‐titration of antiplatelet therapy, shortening DAPT duration, and selecting optimal single‐agent strategies [287, 288]. The TALOS‐AMI trial demonstrated that, for STEMI or NSTEM patients undergoing their first PCI, clopidogrel plus aspirin as a de‐escalation strategy significantly reduced the risk of net clinical events at 12 months, compared to ticagrelor plus aspirin [289]. Studies such as HOST‐IDEA and TWILIGHT further revealed that short‐term DAPT for 3 to 6 months or even as short as 1 month effectively reduces the risk of bleeding in NSTEMI patients without increasing ischemic events [290, 291, 292]. Regarding single‐agent therapy after DAPT, the TWILIGHT and HOST‐EXAM trials have demonstrated the superiority of clopidogrel and ticagrelor monotherapy, respectively, for long‐term maintenance after PCI in NSTEMI patients [293, 294]. Notably, the STOP‐DAPT3 trial found that in Asian populations, both STEMI and NSTEMI patients experienced an increased risk of stent thrombosis when prasugrel monotherapy was used within 1 month post‐PCI, compared to DAPT. However, further studies are needed to explore the effects of shortening DAPT duration and selecting monotherapy, considering factors such as racial differences, drug metabolism characteristics, and stent types. More robust evidence is required to guide clinical decisions on these strategies [295] (Table 2). Additionally, indobufen, another antiplatelet agent, has garnered significant attention recently. The OPTION trial showed that indobufen significantly reduced the risk of net adverse clinical events at 1 year post‐PCI, compared to aspirin [296]. However, given that this trial excluded a substantial number of ACS patients, the efficacy of indobufen in AMI patients requires validation through additional large‐scale randomized controlled trials (RCTs), potentially expanding the repertoire of effective antiplatelet therapies for AMI.

**TABLE 2:** Ongoing RCTs in AMI treatment.

Trial name	Study population	Key interventions	Follow‐up	Primary outcome
DAN‐DAPT trial [297]	STEMI/NSTEMI; PCI ≤ 72 h; PRECISE‐DAPT score ≥ 25	1.Standard DAPT 2.Genotype‐guided DAPT 3.Abbreviated DAPT	12 months	NACE; bleedings
TACSI trial [298]	ACS; first isolated CABG	ASA + ticagrelor vs. ASA alone	10 years	MACE
TADCLOT trial [299]	STEMI; successful primary PCI	Ticagrel vs. Clopidogrel	≥ 1 month	MACE
Librexia ACS [300]	ACS ≤ 7 days; ≥ 2 risk factors	Milvexian	Up to 3.5 years	CV death, MI, ischemic stroke
REBOOT trial [301]	STEMI/NSTEMI; LVEF>40%; no HF	β‐blocker	Median 2.75 years	Death, non‐fatal reinfarction, HF
DANBLOCK [302]	AMI; LVEF>40%; no HF	β‐blocker	2–4 years	Death, recurrent MI, acute HF, UA, stroke
SHAWN study [303]	ACS; eligible for DES	PCSK9 inhibitor	1 year	MACCEs
OPTION‐STEMI trial [304]	Hemodynamically stable STEMI; multivessel disease	Immediate CR vs. in‐hospital‐staged CR	1 year	Death, non‐fatal MI, unplanned revascularization
RESCUE‐MI study [305]	STEMI; 24–48 h post‐symptom onset	Early invasive strategy vs. conservative strategy	1 month	MI size
CELEBRATE trial [306]	STEMI; planned primary PCI	Zalunfiban	1 year	Ranked 7‐point clinical scale
CLEAN trial [307]	STEMI; primary PCI	Nicorandil	12 months	CV death, nonfatal MI, TVR, unplanned hospitalization
ASV‐AMI trial [308]	AMI; successful primary PCI; hemodynamically stable	SV treatment in 2 h vs. 3–7 days after PCI	≥ 1 year	Echocardiographic, CTR, NT pro‐BNP
EXCELLENT trial [309]	AMI; large infarct; LVEF < 50%	Autologous CD34+ cell therapy	6 months	MACE
HOT‐ AAMI [310]	Anterior STEMI; successful LAD PCI	Hyperoxemic oxygen therapy	12–48 months	Death, HF
Acupuncture ‐STEMI [311]	STEMI post‐PCI	Verum acupuncture	6 months	cTnI level

Abbreviations: ACS, acute coronary syndrome; AMI, acute myocardial infarction; ASA, acetylsalicylic acid; CABG, coronary artery bypass grafting; CR, complete revascularization; CTR, cardiothoracic ratio; CV, cardiovascular; DAPT, dual antiplatelet therapy; DES, drug‐eluting stent; HF, heart failure; LAD, left anterior descending artery; LVEF, left ventricular ejection fraction; MACCEs, major adverse cardiac and cerebrovascular events; MACE, major adverse cardiovascular events; MI, myocardial infarction; NACE, net adverse clinical events; NSTEMI, non‐ST‐segment elevation myocardial infarction; NT‐proBNP, N‐terminal pro‐B‐type natriuretic peptide; PCI, percutaneous coronary intervention; PCSK9, proprotein convertase subtilisin/kexin type 9; STEMI, ST‐segment elevation myocardial infarction; TVR, target vessel revascularization; UA, unstable angina.

#### 5.2.3 Clinical Benefits of β‐Blockers

The role of β‐blockers for secondary prevention post‐AMI, primarily established through landmark trials in the 1980s. In the context of current standard therapies, the role of beta‐blockers in AMI management is being increasingly scrutinized. Early meta‐analyses revealed that beta‐blockers failed to significantly reduce mortality among MI patients in the reperfusion era [312]. More recently, the large RCT REDUCE‐AMI demonstrated that long‐term beta‐blocker therapy in AMI patients who underwent coronary angiography and had an LVEF ≥ 50% did not result in a reduction of the composite primary endpoint of all‐cause mortality or new myocardial infarction [313]. Conversely, the ABYSS trial indicated that discontinuation of long‐term beta‐blocker therapy might increase the risk of cardiovascular hospitalization in MI patients with LVEF ≥ 40% [314]. Therefore, the necessity of long‐term beta‐blocker therapy requires further evaluation, and stratified studies are warranted to identify the optimal target population. The results of several ongoing large RCTs are awaited with interest (Table 2).

#### 5.2.4 Intensity and Timing of Lipid Lowering

The clinical value of intensive lipid‐lowering therapy (LLT) after AMI has been well validated. A greater reduction in LDL‐C is associated with improved CV outcomes and a lower risk of all‐cause mortality in AMI patients [315, 316]. This effect also exists in the elderly population. The FAST‐MI registry study showed that AMI patients aged ≥ 80 years who received high‐intensity statin therapy had a 22% significant reduction in the risk of all‐cause mortality at 5 years, clearly supporting that age should not be a basis for restricting intensive LLT [317]. However, real‐world data show that a considerable proportion of the elderly population still do not use high‐intensity statins, highlighting the gap in clinical practice [318]. In addition, the proportion of AMI patients achieving lipid targets with statin monotherapy remains low in clinical practice. Consequently, combination lipid‐lowering strategies have emerged as a critical approach to optimizing treatment. The IMPROVE‐IT trial demonstrated that the combination of simvastatin and ezetimibe significantly improves cardiovascular outcomes in patients with diabetes or high‐risk non‐diabetic ACS [319]. The recent SWEDEHEART registry study further quantified the importance of timely MI treatment initiation. It demonstrated that early initiation (within 12 weeks post‐discharge) of combined LLT with statins and ezetimibe in AMI patients could result in an additional 0.6% to 1.1% reduction in major adverse cardiovascular events (MACE), compared to delayed combination therapy [320]. The PACMAN‐AMI series of studies elucidated the biological mechanisms of combined therapy through multimodal imaging, demonstrating that adding the PCSK9 inhibitor alirocumab to high‐intensity statin therapy reduces the plaque area volume (PAV) of non‐culprit lesions in AMI patients by 2.13%, decreases the lipid core burden index (LCBI) by 41.24 units, and enhances fibrous cap thickness (FCT) [321, 322]. Regarding clinical outcomes, the ODYSSEY OUTCOMES trial showed that early combination therapy with statins and the PCSK9 inhibitor alirocumab, achieving a transient LDL‐C reduction to < 0.39 mmol/L followed by monotherapy maintenance in ACS patients, significantly reduced long‐term MACE risk by 28%, underscoring the sustained benefits of the “early intensification” strategy [273]. While RCT evidence continues to evolve, future research should focus on delineating the safety thresholds and individualized treatment pathways for long‐term intensive LLT (Table 2).

#### 5.2.5 Other Treatment Strategies

In recent years, diversified interventional strategies targeting the pathophysiological mechanisms of AMI have continued to emerge. Mitochondrial dysfunction, recognized as one of the central mechanisms of myocardial injury, has become a novel therapeutic focus. Basic experimental studies have demonstrated cardioprotective effects and mitigation of reperfusion injury through mitochondrial transplantation [19, 323], targeted delivery of antioxidants [324], and modulation of mitochondrial fusion–fission dynamics [325, 326], thereby promoting myocardial repair. At the same time, anti‐inflammatory therapy directed against residual inflammation after AMI has shown significant value in secondary prevention. Large‐scale randomized clinical trials have confirmed that low‐dose colchicine can effectively reduce the risk of cardiovascular events after AMI [327]. OCT studies further revealed that part of its mechanism of action involves stabilization of coronary plaques [328]. However, the optimal timing of colchicine initiation and its long‐term benefits remain under active investigation. In addition, the monoclonal antibody canakinumab, which targets IL‐1β, has been shown to significantly reduce the risk of recurrent events following AMI, highlighting the therapeutic potential of selective inflammatory modulation in delaying atherothrombosis [329]. Nonetheless, further clinical trials are required to validate its translational value.

In the field of myocardial repair and regeneration, stem cell therapies and bioengineered cardiac patches that combine biomaterials with cells or bioactive factors have continued to advance, showing promise in improving cardiac function, promoting angiogenesis, and reducing fibrotic scarring [12, 330, 331, 332, 333]. Nanotechnology has further refined and optimized AMI treatment at a subcellular level. Nanoparticles (NPs) can achieve precise targeting and controlled release of AMI treatment compounds; newly developed ultrasmall Ptlr bimetallic nanoenzymes can modulate mitochondrial function and inflammatory responses to reshape the myocardial infarction microenvironment, thereby repairing damaged myocardium and improving cardiac function, offering new strategies for myocardial protection and real‐time monitoring [334]. Gene therapy has also garnered increasing attention in AMI, with notable progress at the preclinical stage. The combination of CRISPR‐Cas9 gene‐editing technology and targeted delivery systems has successfully modified key genes such as TLR4, miR‐34a, and CaMKIIδ, resulting in improved cardiac repair following AMI [13, 335, 336, 337]. However, the translation of these therapies into clinical practice still faces major challenges, including complex regulatory approval processes, concerns regarding long‐term safety and efficacy, and barriers to large‐scale clinical implementation. To date, the world's first CRISPR‐based human clinical trials have focused primarily on oncology [338], while further safety and feasibility data are needed to support clinical application in AMI.

TCM has demonstrated unique therapeutic value in the treatment of AMI. The large‐scale RCT China Tongxinluo Study for Myocardial Protection in Patients With Acute Myocardial Infarction (CTS‐AMI) confirmed that Tongxinluo capsules, as an adjunctive therapy beyond guideline‐directed management for ST‐segment elevation myocardial infarction (STEMI), significantly reduce major adverse cardiac and cerebrovascular events (MACCE) in STEMI patients at both 30 days and 1 year [339]. Additionally, “chronotherapy” is emerging as the fourth dimension of precision treatment for AMI. A recent *Nature* study revealed that the core circadian protein BMAL1 and hypoxia‐inducible factor hypoxia‐inducible factor 2 alpha (HIF2A) form a heterodimer in ischemic myocardium, regulating circadian rhythm‐dependent myocardial injury. Based on these findings, a “time‐optimized interventions” strategy was proposed: administering nobiletin (NOB) at night (the trough period of the biological clock) reduces infarct size, while using the HIF2A agonist vadadustat during the day (peak period) enhances ejection fraction and improves cardiac function, offering a new direction for time‐optimized precision treatment of AMI [340].

## 6 Conclusion and Perspective

Research on AMI has expanded from a narrow focus on myocardial injury alone to encompass the entire spectrum of myocardial damage across the disease continuum, as well as its systemic impact on multiple organs. Advances in technology have laid the foundation for mechanistic exploration. Single‐cell and spatial multi‐omics approaches provide unprecedented high‐resolution insights into the cellular heterogeneity of cardiac tissue after AMI, elucidate the dynamic evolution of injury sites, and identify key signaling networks that drive cardiac remodeling. Progress in molecular imaging further enables visualization and dynamic monitoring of the molecular mechanisms underlying culprit lesions, offering new perspectives for risk assessment and longitudinal surveillance. Together, these technologies have revealed the complex spatiotemporal mechanisms of myocardial injury and repair, thereby establishing a molecular basis for the discovery of precision therapeutic targets, prediction of individualized disease trajectories, and development of novel treatment strategies.

Nevertheless, a considerable gap remains between mechanistic discoveries and clinical practice. The development of innovative nanomaterials has facilitated the creation of precision tracers targeting diverse cellular and molecular processes such as inflammation and apoptosis, holding promise for bridging this translational divide.Chronotherapeutics, by elucidating the circadian patterns of AMI pathophysiology, may help optimize the timing of interventions and thereby enhance therapeutic efficacy. TCM has also demonstrated significant benefits in AMI management, with several high‐quality studies supporting its therapeutic efficacy in this context. Furthermore, the impact of AMI extends beyond direct cardiac injury, as its interactions with extracardiac organs are complex and far‐reaching. These systemic consequences remain insufficiently understood and call for deeper investigation. Addressing this challenge requires multidisciplinary collaboration spanning molecular mechanisms to clinical strategies, as well as the adoption of a holistic “pan‐cardiovascular” management paradigm, in order to construct a systematic and precise framework for AMI prevention and control.

## Author Contributions

Y.L. contributed to the topic design, revised the manuscript, and made the decision to submit it for publication. M.Z., Y.L., and Q.X. drafted the initial version of the manuscript. M.Z., Y.L., W.W., and Y.L. revised the manuscript. M.Z. and Y.L. contributed equally to this work. All authors approved the final version of the manuscript.

## Ethics Statement

The authors have nothing to report.

## Conflicts of Interest

The authors declare no conflicts of interest.

## Data Availability

The authors have nothing to report.
